# Prevalence of congenital defects including selected neural tube defects in Nepal: results from a health survey

**DOI:** 10.1186/s12887-015-0453-1

**Published:** 2015-09-21

**Authors:** Shiva Bhandari, Jamuna Tamrakar Sayami, Ricky Raj K.C., Megha Raj Banjara

**Affiliations:** Multivitamin-mineral Supplementation Project, Health Resources Consultancy Pvt. Ltd., Kuleshwor, Kathmandu-14, GPO Box: 883, Nepal; National Center for Health Professions Education, Institute of Medicine, Tribhuvan University, Maharajgunj, Kathmandu, Nepal; Central Department of Microbiology, Tribhuvan University, Kirtipur, Kathmandu, Nepal

**Keywords:** Children, Congenital defects, Nepal, Neural tube defects

## Abstract

**Background:**

In resource-limited nations like Nepal, congenital defects, including neural tube defects (NTDs), have great public health impact. NTDs and a few other congenital defects can be prevented by micronutrient supplementation. Without proper research regarding such defects, it is difficult to assess the damage made to health and productivity. This study aims to investigate different congenital defects among children in Nepal.

**Methods:**

Household surveys and health camps were conducted from 2011 to 2012. Physical examination of women of reproductive age (15 to 49 years) was done in selected Village Development Committees of nine districts in three ecological regions of Nepal. Congenital defects, including NTDs, were examined in children (age 0 to 5 years) who were alive at the time of the survey. Data entry and analysis was performed by using SPSS version 11.5.

**Results:**

21,111 women were interviewed and 27,201 children born to them were assessed. The prevalence of congenital defects was 52.0 (95 % CI: 44.0–61.0) per 10,000 children. The prevalence of selected NTDs was 4.0 (95 % CI: 2.0–7.0) per 10,000 children. Among the neural tube defects, encephalocele, myelomeningocele and dermal sinus were the major ones, having almost the same prevalence in the Hill and Terai regions. The majority of children with genital abnormalities (17.0 per 10,000 children; 95 % CI: 10.0–28.0) and limb deformities (14.0 per 10,000 children; 95 % CI: 8.0–24.0) were found in the Terai. The rate of congenital birth defects was higher in the regions where women were in poor health.

**Conclusion:**

There is high prevalence of congenital defects in Nepal. Since such defects add a burden to families and society, it is imperative that health policies addressing programs like supplementation, fortification and dietary diversification be implemented.

## Background

Congenital defects (birth defects) are structural or functional anomalies, which are present at the time of birth. Globally, congenital defects affect an estimated 1 in 33 infants and result in approximately 3.2 million birth defect-related disabilities every year [1]. Congenital defects may result in long-term disability, which may have significant impacts on individuals, families, health-care systems and societies. Although congenital defects may be genetic, infectious or environmental in origin, many can be prevented. One of the most severe defects that can be prevented is neural tube defects (NTDs). NTDs are serious birth defects of the brain and spine, which can be prevented by the supply of folic acid before and during pregnancy [2]. They happen very early in pregnancy when the neural tube, which becomes the brain and the spine, does not close properly, and they are a major cause of death and lifelong disability. Each year more than 300,000 babies are born with neural tube defects worldwide [3] of which almost 70 % are from low- and middle-resource countries. Increasing folic acid intake among women of reproductive age can help prevent these defects [4]. Even in developed nations like the United States, 3000 pregnancies are affected by NTDs every year [5]. The total lifetime direct cost of care for a child born with NTDs is very high as shown by a study in United States [6].

In Nepal, there is limited information regarding the prevalence of congenital defects including NTDs. Without proper research regarding such defects, it is difficult to assess the damage made to health and productivity. Therefore, the aim of this study was to determine the prevalence of congenital defects and selected NTDs in Nepal.

## Methods

### Study area

Nepal is divided into three distinct ecological regions: Mountain, Hill and Terai. The Mountain region has rocky terrain and lies to the north. It has only about 7 % of the total population. In the middle lies the Hill region, which occupies 42 % of the total land area. About 43 % of the total population lives in the Hill region. The Terai region lies in the southern part of the country and has flat terrain. While it constitutes only 23 % of the total land area in Nepal, 50 % of the population live here. Each region is subdivided into districts and within the districts are Village Development Committees (VDCs). In consultation with the Nepal Ministry of Health and Population, and according to feasibility of the research work, nine districts (Dolakha-Mountain; Illam, Kavrepalanchowk, Kathmandu, Lamjung and Kaski-Hill; Sarlahi, Nawalparasi and Kailali-Terai) covering three ecological regions of Nepal were included. From nine districts, four to eight VDCs were selected in consultation with District Public/Health Offices.

### Study population

Children aged 0 to 5 years who were alive at the time of the survey and their mothers residing in the selected geographical areas were included in the study.

### Study design and health camp

Both household surveys and health research camps were conducted from 2011 to 2012. Interviews of reproductive age women were done in the household survey using structured questionnaires and pictures of different types of defects. The women were invited to attend a health camp by the field supervisor at the time of survey. The health camps were conducted in the health facilities of the concerned VDCs for clinical assessment. A qualified doctor conducted physical examinations of women and children. If the mothers were not able to bring their children for some reason, they were shown pictures of malformations and asked to indicate if their child had those malformations. Nurse and health facility staffs were involved in the assessment of women, which included measuring height, weight, and blood pressure. After the physical examination, capillary blood samples were tested for hematocrit determination by a laboratory technician with a hematocrit machine (Heamata STAT-II, STI Separation Technology Inc., USA). A structured nutrition education program was also conducted, highlighting the requirement of micronutrients for reproductive aged women specifically for the prevention of NTDs and other birth defects.

### Sample size

This study is a part of an intervention study where reproductive age women were supplied multivitamin-minerals for one year. The study was primarily designed to test model of distribution of multivitamin-minerals and to test the education module. Altogether 21,371 women were enrolled in the survey. However, only 21,111 women participated in both the survey and health camps. A total of 27,201 children (3079 in Mountain, 15,156 in Hill and 8966 in Terai) born within the past five years before the study period were included in the study. Not all women were included in the study because of the exclusion of missing values (n = 6), absence of women in the health camps (n = 214) and incomplete information from some (n = 40).

### Ethical approval

Ethical clearance was taken from the Ethical Committee of the Nepal Health Research Council (NHRC reg. no. 5/2011) as per national health research policy. Written consent was obtained from the Ministry of Health and Population and District Public/Health Offices from targeted districts. Written and oral informed consent was taken from the participant women. In addition, written consent from parents/guardians was taken in case of participants under 18 years of age.

### Anthropometric measurements

Weights of the women were measured to the nearest 0.1 kg on a battery powered digital scale (Seca GmBH & Co.kg., Germany) and heights were measured to the nearest centimeter using a height scale following standard anthropometric techniques [7]. For weight and height measurements, study subjects removed their shoes and jackets and wore light clothing. Body mass index (BMI) of the study subjects was calculated by dividing the weight in kilograms by the height in meter squared (kg/m^2^). A BMI less than 18.5 was considered as underweight [8]. Anemia was defined as a hematocrit value less than 35 % and normal as more than 35 % [9].

### Data analysis

Data entry and analysis was performed by using SPSS for windows version 11.5 (SPSS Inc.). Descriptive analysis was done and the result was expressed in percentage, ratio and rate. The lower and upper limits of the 95 % confidence interval (CI) for a proportion were calculated.

### Operational definitions

- *Prevalence*: The presence of any congenital defects in children (numerator) in the total population of children (0 to 5 years of age) per 10,000 children (denominator).
- *NTD:* Any one of these defects: anencephaly, spina bifida (myelomeningocele and meningocele), encephalocele, dermal sinus and caudal agenesis.
- *Cleft palate*: An opening in the roof of the mouth due to a failure of the palatal shelves to come fully together from either side of the mouth.
- *Cleft lip*: A fissure in the upper lip that is due to failure of the left and right sides of the fetal lip tissue to fuse.
- *Limb deformities*: Any structural or functional damage of the hands or legs at birth.
- *Genital abnormalities*: Any physical abnormality of the male or female internal or external genitalia present at birth.
- *Any other deformities*: Any type of deformities other than mentioned above (for example, outgrowth of skin mass from head scalp).

## Results

The prevalence of congenital defects was 52.0 per 10,000 children while the prevalence of selected NTDs was 4.0 per 10,000 children. The prevalence of congenital defects was higher (58.0 per 10,000 children) in the Terai region (Table 1).

**Table 1:** Prevalence of neural tube and other birth defects

Indicators	Prevalence per 10,000 (n/N)*	95 % CI
Prevalence of congenital defects	52.0 (142/27201)	44.0–61.0
Prevalence of selected NTDs only	4.0 (11/27201)	2.0–7.0
Prevalence of other congenital defects except NTDs	48.0 (131/27201)	40.0–57.0
Prevalence of congenital defects in Mountain	16.0 (5/3079)	7.0–38.0
Prevalence of congenital defects in Hill	56.0 (85/15156)	45.0–69.0
Prevalence of congenital defects in Terai	58.0 (52/8966)	44.0–76.0

*Abbreviations*: *CI* confidence interval

* n denotes the children with congenital defects and N denotes total live children

Among the neural tube defects, encephalocele, myelomeningocele and dermal sinus were the ones having almost the same prevalence in the Hill and Terai regions. However, in the Mountain region no such defects were recorded (Table 2).

**Table 2:** Type of neural tube defects

Neural tube defects	Hill	95 % CI	Terai	95 % CI
	n(prevalence per 10,000)		n(prevalence per 10,000)	
Encephalocele	4 (3.0)	1.0–7.0	4 (4.0)	1.0–11.0
Myelomeningocele	1 (1.0)	0.0–4.0	1 (1.0)	0.0–6.0
Dermal sinus	0 (0.0)	–	1 (1.0)	0.0–6.0

*Abbreviations*: *CI* confidence interval

The majority of children with genital abnormalities (17.0 per 10,000 children) and limb deformities (14.0 per 10,000 children) were found higher in the Terai. Any other deformities were found higher (21.0 per 10,000 children) in the hilly districts (Table 3).

**Table 3:** Type of other congenital defects except NTDs

Birth defects	Mountain n(prevalence per 10,000)	95 % CI	Hill	95 % CI	Terai	95 % CI
			n(prevalence per 10,000)		n(prevalence per 10,000)	
Hydrocephalus	0 (0.0)	–	8 (5.0)	2.0–10.0	8 (9.0)	5.0–18.0
Scoliosis	0 (0.0)	–	1 (1.0)	0.0–4.0	1 (1.0)	0.0–6.0
Cleft palate	2 (6.0)	2.0–23.0	7 (5.0)	2.0–10.0	4 (4.0)	1.0–11.0
Cleft lip	0 (0.0)	–	3 (2.0)	1.0–6.0	1 (1.0)	0.0–6.0
Limb deformities	0 (0.0)	–	19 (13.0)	8.0–20.0	13 (14.0)	8.0–24.0
Genital abnormalities	0 (0.0)	–	11 (7.0)	4.0–13.0	15 (17.0)	10.0–28.0
Any other deformities	3 (10.0)	3.0–29.0	31 (21.0)	14.0–28.0	4 (4.0)	1.0–11.0

*Abbreviations*: *CI* confidence interval

The rate of congenital birth defects was higher in the regions where there was poor health status of women as indicated by low BMI and high anemia rate. However, there was no such relation in the regions with women with hypertension (Table 4).

**Table 4:** Ecological relations of congenital defects with general health of women

Ecological regions	Anemia, n (%)	Underweight BMI, n (%)	Hypertension, n (%)	Congenital defects per 10,000
Mountain	20 (2.2)	54 (6.1)	85 (9.4)	16.0
Hill	2165 (26.0)	1161 (14.8)	432 (5.2)	56.0
Terai	1232 (24.7)	1235 (26.6)	431 (8.6)	58.0

## Discussion

The present study showed a prevalence of selected NTDs of 4.0 per 10,000 children. The prevalence is underestimated since these results did not include those children who might have died due to NTDs, the majority with anencephaly. In addition, the results were obtained from the women and children who participated in the survey and health camps. The present result is far less than the status of NTDs in India, 17.0 per 10,000 births [10]. This is most likely due to the inclusion of anencephaly cases in the study of India but not in the present study. However, in developed nations like the USA, the estimates are below 3.0 per 10,000 births [11] and in Australia below 5.0 per 10,000 births [12]. Even in a study done in Iran, the prevalence of NTDs is below 10.0 per 10,000 live births [13]. The reduction in the frequency of NTDs is mainly due to the supplementation of folic acid before pregnancy, which is still lacking in Nepal. Although the government of Nepal supplies free iron and folic acid to pregnant women after the first trimester [14], it is very late, as the formation of the neural tube begins from the 28^th^ day after conception. In addition, most of the pregnancies in Nepal are unplanned and are noticed only a month or two after conception. Therefore, there is a need to review the policy. All women of childbearing age should get free iron/folic acid by either supplementation or fortification throughout their reproductive age.

The congenital defects in the present study varied with the ecological regions: the highest prevalence being in the Terai. Although genetic and environmental factors can cause congenital anomalies [15], poor nutrition can also be one of the most important causative factors. Studies have shown that some of the defects due to lack of proper nutrition can be prevented, thereby saving many lives [16, 17]. An important part of a nutrition program is folic acid supplementation, which can prevent birth defects [18]. In addition, periconception folic acid or folic acid-containing multivitamin supplementation has resulted in a breakthrough in the primary prevention of neural-tube defects, cardiovascular abnormalities and other defects [19].

The prevalence of encephalocele, myelomeningocele and dermal sinus are higher in the Terai region. These defects can mostly be prevented by the proper supplementation of folic acid or fortification of food items with folic acid [16]. In addition, education regarding the benefits of taking folic acid can play an important role [20]. Since very few women were enrolled in the Mountain area, there was no prevalence of NTDs noted. Although there seems to be an absence of NTDs in the Mountain area, we should still provide folic acid supplementation in order to prevent such defects.

This study showed that the overall prevalence of congenital defects was 52.0 per 10,000 children. This is higher than the prevalence of congenital anomalies (27.0 per 10,000 live births) in a study done in the northwest of Iran [13]. Even in developed nations, the prevalence of congenital anomalies is very high- up to 239.0 per 10,000 births [21]. The prevalence of genital abnormalities (17.0 per 10,000 children) and limb deformities (15.0 per 10,000 children) were higher in the Terai in Nepal. There are several causes of congenital defects, the most important being environmental and genetic factors [15], poor nutritional status of mothers, improper drug use during pregnancy, and alcohol use during pregnancy [22]. In addition, lack of proper knowledge regarding prenatal care in mothers can contribute to congenital defects. Therefore, control of environmental risk factors is a crucial policy priority for the primary prevention of congenital defects in the population, including preconception care and whole population approaches.

The present study showed that in the regions where there is poor nutritional health status as shown by low BMI, there was a high number of congenital defects at birth. This implies that poor nutrition can also have a great impact on health [23], making more children disabled and even taking their lives [24]. Consequently, it can add a significant burden to the national economy. The government should be judicious in its plans and actions to reduce under nutrition.

Although the study showed the prevalence of congenital defects in Nepal, it has some limitations. First, detection of birth defects was limited to the most obvious ones that were shown to women through pictures. This would lead to an undercounting of other defects. The NTD prevalence obtained in this survey in Nepal is low for a number of reasons. The current survey failed to capture anencephalic cases that are stillborn or die shortly after birth, and anencephaly may account for half of all NTDs. Prevalence at birth could not be determined as this study was a cross-sectional study of children aged 0 to 5 years that were alive at the time of the survey. Therefore, our prevalence estimates would underestimate the birth prevalence, particularly for defects that are associated with shorter periods of survival. Pregnancy outcomes such as spontaneous abortions, fetal deaths and elective terminations impact the overall rate of NTDs. Finally, use of unwanted drugs, alcohol and other environmental effects that can lead to birth defects were not considered.

## Conclusion

The prevalence of selected NTDs and other congenital defects is high in Nepal. However, some of these defects can be prevented through proper nutrition and either supplementation or fortification of women’s diets with folic acid. It is the responsibility of all concerned people and organizations to rectify this situation.

## Abbreviations

BMI: Body Mass Index
CI: Confidence Interval
NHRC: Nepal Health Research Council
NTDs: Neural tube defects
SPSS: Statistical Package for Social Sciences
VDC: Village Development Committee

## References

[1] World Health Organization. Congenital anomalies. Fact sheet N°370. In: Media centre. World Health Organization, Geneva; 2014. http://www.who.int/mediacentre/factsheets/fs370/en/. Accessed 10 Jan 2015.

[2] Bestwick JP, Huttly WJ, Morris JK, Wald NJ (2014). Prevention of neural tube defects: a cross-sectional study of the uptake of folic acid supplementation in nearly half a million women. PLoS One.

[3] Shibuya K, Murray CJ, Murray CJ, Lopez AD (1998). Congenital anomalies. Health dimensions of sex and reproduction: the global burden of sexually transmitted diseases, HIV, maternal conditions, perinatal disorders, and congenital anomalies.

[4] Centers for Disease Control and Prevention (CDC) (2010). CDC Grand Rounds: additional opportunities to prevent neural tube defects with folic acid fortification. MMWR Morb Mortal Wkly Rep.

[5] Centers for Disease Control and Prevention (CDC) (2004). Spina bifida and anencephaly before and after folic acid mandate-United States, 1995–1996 and 1999–2000. MMWR Morb Mortal Wkly Rep.

[6] Grosse SD, Ouyang L, Collins JS, Green D, Dean JH, Stevenson RE (2008). Economic evaluation of a neural tube defect recurrence-prevention program. Am J Prev Med.

[7] Bruce C (2001). Anthropometric indicators measurement guide.

[8] World Health Organization. Global database on Body Mass Index. BMI classification. World Health Organization, Geneva; 2006. http://apps.who.int/bmi/index.jsp?introPage=intro_3.html. Accessed 10 Jun 2014.

[9] Chernecky CC, Berger BJ (2001). Laboratory Tests and Diagnostic Procedures.

[10] Bhide P, Sagoo GS, Moorthie S, Burton H, Kar A (2013). Systematic review of birth prevalence of neural tube defects in India. Birth Defects Res A Clin Mol Teratol.

[11] Parker SE, Mai CT, Canfield MA, Rickard R, Wang Y, Meyer RE (2010). Updated National Birth Prevalence estimates for selected birth defects in the United States, 2004–2006. Birth Defects Res A Clin Mol Teratol.

[12] Abeywardana S, Sullivan EA (2008). Neural tube defects in Australia. An epidemiological report. Cat. no. PER 45.

[13] Hossein MA, Kargar Maher MH, Afsharnia F, Dastgiri S. Prevalence of congenital anomalies: a community-based study in the Northwest of Iran. ISRN Pediatr. 2014. 10.1155/2014/920940 PMC4005020 24995131

[14] Ministry of Health and Population (MOHP) [Nepal], New ERA, ICF International Inc. Nepal Demographic and Health Survey, 2011. Kathmandu, Nepal: Ministry of Health and Population, New ERA, and ICF International, Calverton, Maryland, 2012.

[15] Padmanabhan R (2006). Etiology, pathogenesis and prevention of neural tube defects. Congenit Anom (Kyoto).

[16] Blencowe H, Cousens S, Modell B, Lawn J (2010). Folic acid to reduce neonatal mortality from neural tube disorders. Int J Epidemiol.

[17] Wilson RD, Davies G, Desilets V, Reid GJ, Summers A, Wyatt P (2003). The use of folic acid for the prevention of neural tube defects and other congenital anomalies. J Obstet Gynaecol Can.

[18] Green NS (2002). Folic acid supplementation and prevention of birth defects. J Nutr.

[19] Czeizel AE, Banhidy F (2011). Vitamin supply in pregnancy for prevention of congenital birth defects. Curr Opin Clin Nutr Metab Care.

[20] Horn F, Sabova L, Pinterova E, Hornova J, Trnka J (2014). Prevention of neural tube defects by folic acid - awareness among women of childbearing age in Slovakia. Bratisl Lek Listy.

[21] Dolk H, Loane M, Garne E (2010). The prevalence of congenital anomalies in Europe. Adv Exp Med Biol.

[22] Francine R, Pascale S, Aline H (2014). Congenital anomalies: prevalence and risk factors. Univ J Public Health.

[23] Dabone C, Delisle HF, Receveur O (2011). Poor nutritional status of schoolchildren in urban and peri-urban areas of Ouagadougou (Burkina Faso). Nutr J.

[24] Black RE, Victora CG, Walker SP, Bhutta ZA, Christian P, de Onis M (2013). Maternal and child undernutrition and overweight in low-income and middle-income countries. Lancet.

